# AI-Based Decision Support System for Traumatic Brain Injury: A Survey

**DOI:** 10.3390/diagnostics13091640

**Published:** 2023-05-05

**Authors:** Flora Rajaei, Shuyang Cheng, Craig A. Williamson, Emily Wittrup, Kayvan Najarian

**Affiliations:** 1 Department of Computational Medicine and Bioinformatics, University of Michigan, Ann Arbor, MI 48109, USA; frajaei@med.umich.edu (F.R.);; 2 Department of Neurosurgery, University of Michigan, Ann Arbor, MI 48109, USA; 3 Max Harry Weil Institute for Critical Care Research and Innovation, University of Michigan, Ann Arbor, MI 48109, USA; 4 Michigan Institute for Data Science, University of Michigan, Ann Arbor, MI 48109, USA; 5 Department of Emergency Medicine, University of Michigan, Ann Arbor, MI 48109, USA; 6 Department of Electrical Engineering and Computer Science, University of Michigan, Ann Arbor, MI 48109, USA; 7 Center for Data-Driven Drug Development and Treatment Assessment (DATA), University of Michigan, Ann Arbor, MI 48109, USA

**Keywords:** traumatic brain injury, artificial intelligence, decision support systems, diagnosis, severity assessment, prognosis

## Abstract

Traumatic brain injury (TBI) is one of the major causes of disability and mortality worldwide. Rapid and precise clinical assessment and decision-making are essential to improve the outcome and the resulting complications. Due to the size and complexity of the data analyzed in TBI cases, computer-aided data processing, analysis, and decision support systems could play an important role. However, developing such systems is challenging due to the heterogeneity of symptoms, varying data quality caused by different spatio-temporal resolutions, and the inherent noise associated with image and signal acquisition. The purpose of this article is to review current advances in developing artificial intelligence-based decision support systems for the diagnosis, severity assessment, and long-term prognosis of TBI complications.

## 1. Introduction

Traumatic Brain Injury (TBI), often referred to as the silent epidemic [1,2,3], is an under-recognized and prevalent public health problem [4,5], with an estimated 64–74 million individuals diagnosed with TBI each year [3]. Motor vehicle crashes, falls, firearm-related suicides, assaults, and high-intensity sports are among the most common causes of TBI [6,7,8]. According to the United States Centers for Disease Control and Prevention (CDC) report in 2020, an estimated 176 Americans die from TBI-related causes every day [9]. Depending on the extent of brain injury, survivors may experience short- to long-term complications including physical, psychological, and cognitive impairments. Changes in the decision-making process, attention deficiency, memory concern, lack of impulse control, increased aggregation, and higher suicidality are observed in individuals with a history of TBI [5,10,11,12] and are heightened if the injury happens during childhood [13,14,15,16]. 

Despite the remarkable progress in understanding brain injury, the development of TBI remains complex which makes the assessment of the patient upon admission and during their hospital stay challenging. The pathophysiology of TBI is often divided into two phases. After the initial physical insult to the brain, irreversible lesions known as the primary injury occurs [17,18]. Then, a series of molecular, metabolic, and inflammatory responses can cause secondary injury that is directly mediated by intracranial hypertension, midline shift (MLS), ischemia, herniation, infarction, and cerebral vasospasm [19,20,21]. Unlike primary injuries, the extent of secondary injuries can be decreased or delayed by rapid and precise injury evaluation, maintaining patient homeostasis, and appropriate medical interventions during the “golden hours” of treatment after the initial brain lesion [22,23].

The injury assessment of TBIs relies on clinical examinations based on the Glasgow Coma Scale (GCS) and pupillary reactivity as well as brain imaging, primarily computed tomography (CT) scans [24,25]. The difficulty with TBI assessment is two-fold. First, in practice, clinicians only use traditional manual measurements derived from CT slices. These examinations are tedious, subject to inter- and intra- observer variability, and fail to completely and accurately extract and quantify important characteristics of the injured area. On the other hand, TBI is a complex, heterogeneous event that, unlike other neurological disorders, may not produce predictable clinical symptoms. Therefore, in order to address both of these difficulties, automated systems that can analyze the massive data associated with TBI patients [23] to quickly and efficiently estimate the extent of brain injury [26] and provide prognostic information are highly in demand. Such systems can potentially standardize the management of TBI patients, reduce the chance of human error, speed up the decision-making process, and personalize medical management based on individual patients’ pathophysiological symptoms and complications. This may be particularly useful in small rural hospitals that often lack expertise in neurosurgery and neurological intensive care, and where there is both a higher incidence of TBI and increased mortality due to TBI [27,28].

The purpose of this paper is to review the past two decades’ efforts in developing computer-aided decision support systems for TBI diagnosis and prognostication and discuss the limitations of current methods that have not allowed them to be implemented into the daily practice of clinicians. The future research direction for advancing such systems is also discussed in the last sections. 

## 2. Hematoma Detection and Quantification

Hematoma is one of the most common sequelae of TBI. Based on its location within or outside of the brain parenchyma, a hematoma can be classified as intra-axial or extra-axial. Extra-axial hematomas include subdural hematomas (SDH), epidural hematomas (EDH), and subarachnoid hemorrhage (SAH). An intra-axial hematoma can variably be termed intraparenchymal hemorrhage (IPH), intracerebral hemorrhage (ICH), or cerebral contusion [29,30]. Previous studies have shown the importance of hematoma type and volume in predicting TBI outcomes [31,32]. Delayed management of the hematoma can lead to raised intracranial pressure, focal neurological dysfunction, unconsciousness, and death. Hematoma quantification on CT slices is classically done by the ABC/2 volume estimation technique which often overestimates the actual hematoma volume [33,34]. Another common approach is computer-assisted planimetric analysis which is very time-consuming [33,34]. Computer-aided image processing techniques can help clinicians to assess hematoma shape and size and speed up the physicians’ decision-making process. Common challenges with these methods are the inherent noise associated with CT scans, obstructions from the skull, ventricles, or soft tissue edema, and variability in hemorrhage location, size, brightness, and pixel intensity. In this regard, a number of brain hemorrhage segmentation, classification, and quantification algorithms have been proposed. The majority of brain hematoma segmentation algorithms can be classified as thresholding, region growing, level-set, active contour, fuzzy c-means (FCM) as well as deep-learning (DL)- and neural-network (NN)-based algorithms. These segmentation algorithms are usually coupled with established classifiers such as support vector machine (SVM), decision tree, k-nearest neighbor (KNN), and the k-means clustering model to classify hematoma versus non-hematoma regions or distinguish different hematoma types. Shahangian et al. [35] used thresholding to segment and detect candidate hematoma regions. In this technique, each pixel of the hemorrhage region is segmented based on the segmentation thresholds that are defined by image intensity distribution. Bardera et al. [36] used a semi-automated approach by manual adjustment of the initialization seed points and threshold values. An intensity-based region-growing method was then used to segment the entire region. This technique detects the hematoma region by identifying the neighboring pixels that have similar grayscales as the starting point. Liao et al. [37] used adaptive thresholding and connectivity information to locate hematoma voxels. A multi-resolution binary level set segmentation algorithm was then applied to distinguish the intracranial hematoma from soft brain tissue. In another approach, Farzaneh et al. [38] used distance regularized level set evolution (DRLSE) to develop an automated SDH segmentation model considering variations in intensity, size, shape, and location of the hematoma region. In order to reduce the complexity of the DRLSE method due to reinitialization, Shahangian et al. [39] proposed a modified distance regularized level-set evolution (MDRLSE) method to detect the hematoma regions. The extracted shape and texture features from the segmented region were then used to detect four different hematoma subtypes. A combination of FCM clustering and a region-based active contour model was employed by Bhadauria et al. [40] to detect ICH. A framework integrating the Gaussian mixture model (GMM) and parameter estimation using expectation maximization was implemented to find the hematoma component [41]. The above-mentioned methods either rely only on intensity values for selecting the region of interest at the first step or are sensitive to the initialization mask. However, due to the variations in imaging protocols and patient conditions, hematoma intensity varies and, in some cases, overlaps with that of normal brain tissue or other anatomical structures such as the straight sinus. 

More recently, deep learning techniques have been applied to acute brain hematoma segmentation and quantification. Nag et al. [42] used an autoencoder network to generate an initialization mask, combined with an active contour Chan-Vase model to segment acute intracranial hematoma. Jain et al. [43] implemented 2D and 3D U-net-based CNN algorithms for brain extraction, hematoma segmentation, and volumetric quantification of intracranial lesions. Chilamkurthy et al. [44] applied a natural language processing (NLP) algorithm on head CT scans combined with clinical reports to detect five different hematoma subtypes. Patel et al. [45] used the Long-Short Term Memory (LSTM) model to extract features from multiple stacked CT slices and detect ICH. This method provides more spatial information compared to models that are based on the single 2D slice analysis. In a similar approach, Grewal et al. [46] implemented a Recurrent Attention DenseNet (RADnet) framework for hemorrhage detection from 3D scans. Chang et al. [47] designed a customized 2D/3D mask region of interest (ROI)-based Convolutional Neural Network (CNN) framework to segment and detect SDH/EDH, SAH, and IPH. Farzeneh et al. [48] used a combination of hand-crafted features and U-net-extracted features [49] to specifically detect and quantify SDH. The proposed model was generalizable to various SDH types with greater than 25 cc of blood. Yao et al. [50] proposed a modified U-net in which dilated convolution was introduced while down-sampling and up-sampling layers were removed. Furthermore, low-level features and high-level features were also combined. The efficiency of this model in improving the prediction resolution and the final segmentation performance over GMM [41] and previous U-net models was demonstrated. Nijiati et al. applied a U-net-based Sym-TransNet framework to segment five hematoma subtypes [51]. Inkeaw et al. [52] adopted a three-dimensional CNN approach [53] to detect three hematoma subtypes. Monterio et al. [54] used a two-step training strategy to develop a CNN-based model to segment, detect, and quantify three different brain hemorrhages. Mansour et al. [55] used optimal image segmentation with the Inception v4 network and developed a DL-based model for ICH detection and classification. One of the major limitations in training hematoma segmentation models is the manual labeling of a huge number of CT scans. In order to overcome this problem, Yao et al. [56] developed a fully automated acute hematoma segmentation model combining active learning and active contour models. Statistical and textural features extracted from superpixels were used to train the proposed model which could achieve a similar outcome with five times less annotated data compared with that of established machine learning (ML) models. Table 1 summarizes the discussed methods for hematoma detection and quantification.

## 3. Intracranial Pressure

According to the Guidelines for the Management of Severe Traumatic Brain Injury [61], increased intracranial pressure (ICP) resulting from edema or hematoma is one of the important causes of secondary brain injury and is associated with poor outcomes in TBI patients. Raised ICP causes reduced cerebral perfusion pressure, brain tissue compression, deformation, and herniation, further complicating the injury [62,63]. Pathologically, clinical interventions are required when ICP persistently rises above 22 mmHg, because values above this level are correlated with an increased risk of mortality [64]. Currently, ICP is assessed invasively by direct measurement of ventricular or parenchymal pressure which could increase the risk of bleeding, infection, and brain tissue damage [65,66,67,68], with current guidelines recommending placement of an invasive ICP monitor for all patients with an abnormal head CT and GCS less than eight. Hematoma volume and shift of the brain’s midline, typically measured at the level of the ventricles, are associated with a higher likelihood of ICP elevation. Since these manual estimations are slow and susceptible to human error, automated non-invasive methods that can quickly, precisely, and consistently predict ICP are preferred. During the past years, several non-invasive methods to estimate ICP have been suggested, which may allow refinement of the selection criteria for invasive monitor placement [65]. These methods can be grouped into physiological-, morphological-, and textural-based methods. Physiological-based methods are based on the physiological changes that are associated with raised ICP such as the arterial blood pressure (ABP) and flow velocity (FV) of major cerebral arteries [69,70]. Morphological-based methods often use imaging techniques to quantify morphological changes, such as optic nerve sheath diameter (ONSD) [65]. The optic nerve is the extension of the central nervous system and is in touch with the brain subarachnoid space. Recently, estimating the increased ICP from measuring the optic nerve has gained interest because it can non-invasively be measured by CT and Magnetic Resonance imaging (MRI) as well as ultrasounds methods [71,72,73,74,75]. Nevertheless, these methods are still based on manual measurement and have not been automated yet. In a different morphological-based approach, Pappu et al. [76] developed a semi-automated method that segments brain parenchyma from cerebrospinal fluid (CSF) and computes the ratio of CSF volume to whole intracranial volume (csfv/icvv) to estimate ICP. However, the efficiency of this model was limited to cases with csfv/icvv > 0.034 and could not distinguish between high and low ICP when the csfv/icvv ratio was smaller than 0.034. Textural-based methods assess subtle changes in tissue density and image texture that are sometimes hidden from human eyes to automate ICP prediction. A combination of features extracted from Fourier analysis, Gray Level Run Length Matrix (GLRLM), Dual Tree Complex Wavelet Transform (DT-CWT), and histogram analysis have been used for ICP level classification in TBI patients [77,78,79,80,81]. It was demonstrated that the energy of different sub-band images of 2D fully anisotropic Morlet wavelet transformations could be used to determine the dominant textural orientation of the brain tissue in TBI patients and was later shown to be more competent than DT-CWT in ICP prediction [82,83].

## 4. Midline Shift

MLS is the measurement of horizontal displacement in the brain structure via imaging modalities. The amount of MLS is correlated with increased ICP [84] and is one of the most informative features in CT classification scoring systems including Marshall and Rotterdam scores [85,86]. Clinicians typically use the attachment of falx cerebri to the skull to determine the ideal midline (iML) [84]. The distance between the iML and a pertinent point on the brain scan such as the septum pellucidum (SP), a narrow boundary between two lateral ventricles, is used to calculate the MLS. However, this subjective measurement of MLS on a single slice is prone to human error and does not account for head rotation and overall tissue displacement. 

Over the past years, a great number of MLS estimation models have been proposed. Liao et al. classified these models into symmetry-based models and landmark-based models [84]. In symmetry-based models, a line connecting all displaced and deformed structures, called the deformed midline (dML), is identified and its distance from the iML is considered the MLS. Liao et al. [87] proposed a symmetry-based method to identify the dML at the Foramen of Monro (FM) level. In this model, the dML consists of the lower and upper straight segments of falx cerebri and a central quadratic Bezier curve-shaped segment reflecting the deformed brain tissue. This method was based on the manual selection and rotation of the single selected brain CT scan and has a lower success rate of MLS measurements in severe cases. Liu et al. [88] used a linear regression-based algorithm (H-MLS) to model the relationship between the hematoma and midline deformation. Visual symmetry information was then used to adjust the predicted deformed midline. Wang et al. [89] developed a model based on the calculation of the MLS ratio over the maximum width of the intracranial space. In this method, a higher weight was assigned to the darker pixels of pre-selected CT scans and used to plot the weighted midline (WML). The distance between the WML and the iML was then used to estimate the brain shift. 

In landmark-based algorithms, specific anatomical structures, often parts of the lateral ventricles, are selected as landmarks to assess MLS. Chen et al. [79] proposed a method for automated iML detection in which a vertical line is passed through the centroid of the image mass then the image is rotated to yield the best symmetry. The falx cerebri and anterior bone protrusion locations are also considered to make the rotation more precise and determine the iML. Hooshmand et al. [90] developed a fully automated approach for CT slice selection, rotation, and ventricular segmentation. A level-set method was then used to quantify the mean distance between the iML and actual midlines on all selected images. However, a major limitation of this method is that by measuring the MLS as a linear shift, other clinically important information, such as shape, volume, and location of the displaced tissue, is ignored. To solve this issue, a volumetric measurement of shifted brain tissue, namely mid-surface shift (MSS), was suggested to quantify the area between the iML and the dML in all selected slices [91]. Using this method, the ratio between the mid-surface volume and the brain volume was calculated and shown to have a significant correlation with patient outcomes at discharge. Recently, a number of DL-based approaches to assess MLS in the injured brain have been suggested. Wei et al. [92] used a regression-based line detection network (RLDN) to assess MLS in the severely deformed brain. Chilamkurthy et al. [44] used the NLP algorithm to detect tissue shifts larger than 5 mm. Nag et al. [93] applied a U-Net algorithm to segment left/right hemispheres. The junction between these two segments was then traced to form the dML. The summary of AI-based methods for ICP estimation and MLS detection/quantification is presented in Table 2.

## 5. Electroencephalogram (EEG)-Based Methods

EEG refers to the standard digital recording of the brain’s electrical activity. Quantitative interpretations of EEG signal (qEEG) and identification of slight changes in the patterns or type of EEG activity have been suggested to be an indication of mild TBI (mTBI) [96,97,98]. One challenge with using EEG recordings in the emergency room or ICU is that these signals are blended with environmental and physiological artifacts and noises such as electrode displacement, muscle and cardiac activity, and blinking that can affect the accuracy of signal detections [96]. The existing preprocessing methods for removing the EEG artifacts have limited performance, mainly due to the oversimplifying assumptions of stationarity of the brain signals and additivity of EEG noises which can adversely affect the quality of features extracted from EEGs such as qEEG [96,97]. Another important limitation in using these signals is the lack of a universal index for TBI diagnosis which makes the qEEG mapping with severity index very challenging [96]. Several predictive models using quantitative EEG parameters have been proposed, but these models all require validation in much larger and more diverse data sets before their use can be considered in routine clinical practice [99].

## 6. TBI Prognostication

Because the majority of patients with traumatic brain injury die as a result of the withdrawal of life-sustaining treatments due to perceived poor prognosis, accurate prognostication of TBI outcomes is extremely important in determining outcomes and making individualized treatment decisions [100,101]. While outcome prediction is clinically important for the personalized management of TBI, a sufficiently accurate scoring system that captures the heterogeneity of traumatic brain injury remains unavailable. There are several well-validated scoring systems, but they are overly simplistic and fail to incorporate a wide variety of data, particularly imaging data. Advanced computer-aided prognostic ML techniques have been proposed to help clinicians predict early mortality as well as long-term outcomes in patients admitted with TBI. Wang et al. [32] assessed the relative importance of hematoma shape, size, ICH score, and GCS score in predicting 30-day mortality in TBI patients. Abujaber et al. [102] showed that logistic regression (LR) is more efficient than artificial neural networks (ANN) in predicting in-hospital mortality in intubated patients with moderate to severe TBI. Voormolen et al. [103] compared the survival prediction efficiency of the LR algorithm and 22 ML models. Although the AUC of Linear SVM, Quadratic SVM, and Cubic SVM were higher than that of LR in this study, the outcomes were not verified on an external dataset. In another study, Wang et al. [104] demonstrated that XGBoost (XGB) provides better performance than LR algorithms in predicting early mortality in TBI patients. However, considering the higher tendency of XGB to overfit, the generalizability of the selected model needs to be tested on an external dataset. Adil et al. [105] compared shallow neural networks (SNN), deep neural networks (DNN), and elastic-net regularized logistic regression (LRnet) models for TBI prognostication which showed similar prediction performance. In this case, the simpler model, LRnet, would be preferable for its reduced risk of overfitting and also the ease of interpretability and deployment that is critical for the prognostic models. Yao et al. [60] used ICH shape and volumetric-distribution-related features that were obtained from a multi-view CNN model, combined with clinical observations to predict 6-month mortality. The proposed model used a random forest (RF) algorithm and achieved better performance compared to the widely used IMPACT model. Gravesteijn et al. [106] compared the discriminative performance, calibration, and generalizability of established ML models with traditional regression algorithms to predict mortality and unpredictable outcomes using CENTER-TBI and IMPACT-II datasets. It was shown that these outcome performances varied between datasets rather than between the algorithms. According to the above-mentioned studies, no clear difference in performance was observed between regression-based models and ML models and most likely, using novel, more sophisticated algorithms will not improve outcome prediction. Therefore, future studies need to focus on characterizing and incorporating the most informative TBI prognostic predictors. In addition to neuroimaging data, incorporating measures of clinical severity, vital signs, and continuous sensor signals from cardiac telemetry, arterial blood pressure, and ICP monitoring may improve the prediction of neurological deterioration and overall outcomes [107]. One major limitation of integrating ML-based prognosis models in healthcare applications is their lack of interpretability which has greatly affected their adoption in TBI management. To address this issue, Farzaneh et al. [108] proposed a framework for an intelligible prognostic model for TBI outcome prediction six months from hospitalization in which (SHapley Additive exPlanations) SHAP contribution values were used to select the most relevant features followed by clinical expert validation of selected variables. Using this model, each feature returns a contribution at the individual level, and aggregation of all feature contributions determines the ultimate predicted score for each person. In a more recent study, Minoccheri et al. [109] developed a human interpretable neural network model based on tropical geometry and fuzzy logic. Using available information at the time of admission, this model could predict negative outcomes in TBI patients six months after hospitalization. Not only was the classification performance of this intelligible model comparable with established ML models such as SVM, XGB, and RF, but it also allowed extracting rules that only involve a few factors that can be used in isolation if some of the variables are not available. Table 3 shows the summary of reviewed computer-aided decision support systems for TBI prognostication.

## 7. TBI Datasets

The Federal Interagency Traumatic Brain Injury Research Informatics System (FITBIR) [110], founded by the National Institute of Health (NIH) and the Department of Defense (DoD), is a centralized TBI dataset repository that has provided an invaluable platform to share standardized raw data including clinical assessment, medical imaging, and environmental and behavioral history from different studies and clinical trials. This platform serves as a link to current databases, facilitates collaboration between laboratories, and provides a valuable opportunity to share methodologies and related tools. Integration, re-aggregation, and re-analysis of the shared data through this rich, diverse, and high-quality TBI dataset repository can accelerate TBI research progress by identifying targeted and more specific clinical treatments.

Among core FITBIR datasets is the Transforming Research and Clinical Knowledge in TBI (TRACK-TBI) [111]. TRACK-TBI, founded by the National Institute of Neurological and Communicative Disorders and Stroke (NINDS), is a dataset containing standard clinical data from 3000 subjects at 18 U.S. sites. This dataset includes blood biospecimens, CT/MRI imaging, and detailed clinical outcomes across military, sports, and civilian populations. 

MIMIC-III (Medical Information Mart for Intensive Care–III) [112] and eICU (Philips eICU Collaborative Research Database) [113] are also two big publicly available general ICU datasets that include a large number of TBI patients. These datasets were generated from de-identified electronic health records of patients admitted to the ICU. MIMIC-III is a single-center dataset, containing 53,423 ICU admissions between 2001 and 2012, for patients aged 16 years old and older. eICU is a multi-center dataset that consists of 200,859 ICU admissions between 2014 and 2015 for 139,367 unique patients.

## 8. Discussion

TBI is a heterogenous condition that results in a wide spectrum of injuries in terms of symptoms, severity, and outcome. Over the past two decades, there has been a plethora of research on developing automated clinical decision-support systems that can detect and quantify the imaging features of TBI, analyze the brain status and patients’ homeostasis in ICU, assess the injury severity, guide clinical diagnosis, and predict patients’ outcome.

Despite the great potential of AI-based decision support systems in TBI, there is still a noticeable gap between the available technology and clinical practice, which is also reflected in some limitations of this survey.

Apart from a brief section on EEG data, most of the reviewed methods are based on still image data such as CT, MRI, and ultrasound. This is because, beside clinical examinations, current practice in TBI diagnosis primarily employs neuroimaging techniques. Furthermore, the rapid development of computer vision, especially deep-learning-based algorithms in the past decade, has outperformed other data modalities by a considerable margin, thus heavily influencing the scope of this survey.

In sharp contrast to the great number of automated clinical decision support systems and ML algorithms presented in this survey, their performance has not been validated and compared in a uniform manner. This is due to a lack of publicly available benchmark datasets for TBI, especially a large amount of imaging data with annotations provided by clinical experts, which are vital for the training of most deep-learning-based computer vision algorithms.

Therefore, the current state of potential applications of AI-based decision support systems in TBI calls for more collaboration within the field of TBI research, both between the clinical and AI research communities, and within different aspects of TBI such as various types of data and feature modalities, pathology, and severity spectrums.

The most urgent issue is the development of TBI-focused AI research platforms and benchmark datasets which require a lot of effort from the clinical side of the TBI research community. This will facilitate researchers by providing them with large training, validation, and testing datasets, especially a sufficient quantity of annotated images for deep-learning-based computer vision models and other sophisticated models which have a higher chance of overfitting. In this regard, FITBIR provides an invaluable platform to share the standard raw data, methodologies, and related tools.

Most of the existing methods for TBI severity assessment are developed for a specific pathology (e.g., including brain swelling, hemorrhage, raised intracranial pressure, and midline shift) or severity group of TBI patients (e.g., severe or severe to moderate TBI). Future approaches need to be more inclusive, considering all TBI pathologies and severity categories. A hierarchical framework with multiple models that covers the full TBI severity spectrum is recommended.

Although neuroimaging-based methods provide an excellent structural and functional mapping of TBI, they are limited to momentary views of the dynamically evolving brain injury and may fail to detect mild to moderate injuries [96]. In contrast, qEEG-based techniques provide a good temporal and spatial resolution of the brain activities and are promising in detecting non-severe TBI cases [96]. EEG is a promising portable technology that can provide useful information about TBI as soon as it happens. This is particularly important in the rapid, initial diagnosis of at-risk populations such as athletes and military service members. The accuracy of initial detection in TBI-diagnosed subjects can further be validated using neuroimaging techniques as soon as they are transferred to the healthcare units.

Current advances in developing TBI prognostication models suggest that developing complicated models will not drastically improve the injury outcome prediction so future studies need to focus on identifying the most informative variables. Intelligible feature selection methods such as SHAP that return the contribution value of each variable can enable researchers to select the most relevant features. Moreover, adding another layer of validation from clinical experts on TBI on the selected features is necessary for the adoption of the developed model in the clinical domain. 

Another major problem of many ML models with clinical applications is their “black box” essence with limited understandability and transparency. Ideally, a rule-based model with explainable rules aligned with clinical domain knowledge should be considered. This will improve the acceptability of the outputs and facilitate the integration of the developed decision support system into the medical management of TBI. In this regard, using FNN-based models that allow extracting human interpretable data as “if-then” rules would be helpful. These models allow the integration of clinical domain knowledge into the learning process which improves model performance and reduces the amount of required training data. Furthermore, it provides the advantage of rule extraction from a few factors when some of the variables are not available.

## 9. Conclusions

Given the heterogeneity of TBI, future computer-aided decision support systems should be more comprehensive, covering different TBI severity spectrums and abnormalities. This model needs to be validated in multiple settings, including small, rural hospitals that are less likely to participate in research. Because the prognostic information given to families plays a major role in determining prognosis, mediated by the withdrawal of life-sustaining treatment, predictive models must be highly accurate before they can confidently be deployed in clinical practice. Ideally, the logic of their predictions can be understood and interpreted by individual clinicians, which will aid in adoption into clinical practice.

## Figures and Tables

**Table 1:** Summary of different models for hematoma detection and quantification.

Ref.	ML/DL	Algorithm/Method	Dataset Size	TBI-Related Clinical Assessment	Performance	Main contribution
[36]	ML	Intensity-based, region growing algorithm	18	Hematoma segmentation and quantification	Mean matching ratio: 0.80 Mean correspondence ratio: 0.74	Proposing a semi-automated method for hematoma detection and voxel-wise volume estimation
[37]	ML	Multiresolution binary level set method	15	Hematoma segmentation and quantification	Mean Sen: 0.87 Mean precision: 0.89	Automated method, Using adaptive threshold as initial values for binary level set method
[40]	ML	FCM clustering, region based active contour	20	ICH segmentation	Dice coefficient:0.87 Jaccard index: 0.78 Sen: 0.79 Spec: 0.99	The level-set method used by active contour does not need re-initialization and converges faster.
[35]	ML	Thresholding for segmentation, GA-based feature selection, NN classification	_	EDH, ICH, SDH detection	Segmentation Acc: EDH: 0.96 ICH: 0.95 SDH: 0.90 ICH detection and classification Acc: 0.90	Proposing independent hematoma segmentation and classification approach
[41]	ML	GMM, expansion maximization algorithm	11	ICH segmentation	_	Developing a GMM-based model to remove skull and image’s artifacts and detect hematoma
[39]	ML	MDRLSE, hierarchical classifier (using pixel intensity and then SVM classifier)	627	ICH, SDH, EDH IVH detection	Segmentation Acc First classifier: 0.9 Second classifier: 0.94	Using a hierarchical classifier to first classify the IVH from the normal class and then SDH, ICH, and EDH
[38]	ML	DRLSE, Tree bagger classifier	42	SDH detection	AUC: 0.87 Sen: 0.85 Spec: 0.73	Proposing a method for 3D segmentation of SDH considering geometric, textural, and statistical features
[50]	DL	Dilated CNN	62	Hematoma segmentation	Dice: 0.62 Acc: 0.95	Hematoma detection using an FCN model combined with dilated convolutions
[44]	DL	NLP	313,318 (Qure25k dataset); 491 (CQ500 dataset) as validation set	SDH, SAH, IVH, IPH and extradural hematoma detection	AUC ICH: 0.94 Intraparenchymal: 0.95 Intraventricular: 0.93 SDH: 0.95 Extradural: 0.97 Subarachnoid: 0.96	Developing a DL model to detect five different subtypes of intracranial hematoma, cranial vault fractures, mass effect, and midline shift
[46]	DL	Original DenseNet, attention mechanism, RNN	329	Acute hematoma detection	Acc: 0.818 Recall: 0.886 F1-score: 0.847	A combination of CNN and LSTM was used to model 3D CT labeling for brain hemorrhage detection that was benchmarked against specialist clinician.
[47]	DL	Custom 2D/3D mask Region of interest-based CNN	11,021	IPH, EDH/SDH, SAH detection andquantification	Dice: EDH/SDH: 0.86 IPH: 0.93 SAH: 0.77 Pearson correlation coefficient for volume estimation: EDH/SDH: 0.98 IPH: 0.99 SAH: 0.95	A custom model, extracted from the feature pyramid network [57] was implemented for hematoma segmentation, classification, and volume measurement
[56]	ML	Active learning to train SVM classifier, active contour	62	Hematoma segmentation	Dice: 0.55 Acc: 0.97	The proposed model could achieve a comparable result with 5 times less labeled data compared with established ML models.
[42]	DL	Fuzzy-based intensifier, Autoencoder, active contour Chan-Vase model	48	Hematoma segmentation	Dice similarity score: 0.70±0.12 Jaccard index: 0.55±0.14	Implementing unsupervised NN-based method for acute hematoma segmentation
[43]	DL	U-net based CNN	144 subjects from CENTER -TBI and NCT02210221 datasets [58,59]	Hematoma segmentation, volume estimation	Segmentation Dice: 0.697 Volume estimation correlation coefficient: 0.966	Proposing a novel Multi-view CNN with a mixed loss forhematoma segmentation and quantification
[48]	ML/DL	Level set method, U-net, RF	110	SDH segmentation and severity estimation by hematoma volume classification (0–25 cc vs. >25 cc)	Sen: 0.78 Precision: 0.76 DSC: 0.75	Integrating classical image processing methods and DL model to improve the average performance of hematoma detection andquantification
[54]	DL	CNN	937 (CENTER -TBI and NCT02210221) [58,59]; Validation: 500	IPH, EAH, IVH, and perilesional oedema segmentation, detection, and volume quantification	AUC for classification of lesions greater than 0 mL: IPH: 0.87 EAH: 0.89 IVH: 0.89 Perilesional oedema: 0.89	Proposing a CNN-based algorithm for voxel-wise segmentation, detection, and quantification of various TBI lesions and perilesional oedema
[60]	DL	Multi-view CNN	120	ICH detection and volumetric quantification	Dice coefficient: 0.697 ICC: 0.966	Developing a multi-view CNN with dilated convolution and mixed loss to reduce the model sensitivity to the noise and minor shape changes.
[55]	DL	Kapur’s thresholding, EHO algorithm, Inception v4 network, multilayer perception	82	ICH detection and classification	Acc: 0.941 Precision: 0.944 Spec: 0.948 Sen: 0.926	Developing DL-ICH model for image preprocessing, ICH segmentation, feature extraction andclassification
[51]	DL	Sym-TransNet	1357	IPH, IVH, EDH, SDH and, SAH segmentation	Dice coefficient: IPH: 0.78 IVH: 0.68 EDH: 0.359 SDH: 0.534 SAH: 0.337 Five-class: 0.716±0.031	Proposing a U-net based model to detect five different hematoma subtypes
[45]	DL	LSTM	Training and testing: 1554, validation: 386	ICH detection	AUC: 0.96	Combining CNN and RNN to form a bidirectional LSTM model for intracranial hemorrhage detection
[52]	DL	3D CNN, region growing algorithm	153	SDH, EDH, IPH segmentation	Median DSC SDH: 0.48 EDH: 0.71 IPH: 0.37 ICH: 0.59	Developing a hematoma segmentation approach using DL model with 4 different parallel pathways

**Table 2:** Summary of different approaches for ICP estimation and MLS quantification.

Ref.	ML/DL	Algorithm/Method	Dataset Size	TBI-related Clinical Assessment	Performance	Main Contribution
[77]	ML	ReliefF feature selection, SVM	17	ICP > 15 vs. ICP ≤ 15	Acc: 81.79 ± 2.3 Sen: 82.25 ± 1.7 Spec: 81.20 ± 0.04	Textural-based ICP estimation using Histogram analysis, GLRLM, DWPT, Fourier Transform, and DT-CWT
[80]	ML	SVM	17I	ICP > 12 vs. ICP ≤ 12	Acc: 70.2 ± 4.5 Sen: 65.2 ± 8.6 Spec: 73.7 ± 4.6	Textural-based ICP estimation using Histogram analysis, GLRLM, DWPT, Fourier Transform, DT-CWT as well as hematoma volume, manual MLS measurement, age, and ISS
[81]	ML`	SVM	17	ICP > 12 vs. ICP ≤ 12	Acc: 73.7 Sen: 68.6 Spec: 76.6	Textural-based; improving previous model [80] by adding intracranial air cavities, ventricle size related feature
[83]	ML	GA-SVM and GA-KNN based feature selection, SVM classification method	59	ICP > 15 vs. ICP < 15	Acc: 86.5	Textural-based; using anisotropic complex wavelet as textural feature for ICP classification
[82]	ML	GA-SVM based feature selection, SVR classification method	59	ICP > 15 vs. ICP < 15	Acc: 0.94 MAE: 4.25 mmHg	Textural-based; comparing anisotropic complex wavelet transform extracted features vs. DT-CWT extracted features in ICP classification.
[76]		Hounsfield units thresholding	20	ICP > 20 vs. ICP < 20	Acc: 0.67	Morphological-based; brain parenchyma segmentation, ICP estimation based on csfv/icvv ratio
[69]	ML	Black box model	11	ICP	MAE: 4.0 ± 1.8 mmHg	Physiological-based; non-invasive prediction of ICP using ABP and FV of major cerebral arteries.
[70]	ML	SVM	446	ICP	Mean ICP error: 6.7 mmHg	Physiological-based; noninvasive measurement of ICP using ABP and FV of major cerebral arteries
[71]	ML	Linear regression	74	ICP > 20 vs. ICP ≤ 20	AUC: 0.94	Morphological-based; estimating the probability of increase ICP by measuring MRI-based ONSD
[80]	ML	Gaussian mixture model	57	ICP > 12 vs. ICP ≤ 12	Acc: 0.70	Textural-based ICP estimation; using tissue textural features, manual MLS quantification, ISS, and age
[79]	ML	Information gain ratio, GA feature selection, SVM	57	ICP > 12 vs. ICP ≤ 12	Acc: 0.70 Sen: 0.65 Spec: 0.73	Textural-based ICP estimation; automated iML detection; using textural features, MLS, and blood amount to estimate ICP
[87]		Bezier curve, GA	81	MLS	Acc: 0.95	Symmetry-based; detecting dML at the foramen of Monro level; Low performance in case of severe TBI
[88]	ML	H-MLS (Linear Regression based), visual symmetry information	11	MLS	-	Symmetry-based; tracing dML based on the brain hemorrhage detection
[89]		Weighted midline, maximum distance	41	MLS	Acc: 0.92	Symmetry-based; estimating MLS based on the maximum distance between WML and iML close to the foramen of Monro
[94]	ML	CT density, spatial filtering, cluster analysis	273	MLS	Sen:1 Spec: 0.98	Landmark-based; using spatial filtering, CT density thresholds, and cluster analysis to segment blood and CSF. The symmetry of CSF pixels in the lateral ventricles is used to assess MLS
[90]	ML	Level-set	170	MLS	Acc: 0.68	Landmark-based; automating CT slice selection, rotation, and segmentation
[91]	ML	Active contour, Logistic regression	48	MSS vs. MLS	AUC: 0.71 Acc: 0.79	Landmark-based; volumetric measurement of displaced brain mass was significantly correlated with GOS on discharge
[44]	DL	NPL	313, 318 (Qure25k dataset); 491 (CQ500 dataset) as validation set	MLS	AUC: 0·969 Sen: 0.938 Spec: 0.907	Detecting mass effect which consists of MLS, ventricular effacement, herniation, or local mass effect
[92]	DL	RLDN	189 (CQ500 dataset [44] and local resources)	MLS	F1-score: 0.78	Developing a multi-scale bidirectional FCN based method [95] for midline delineation in severe brain deformation
[93]	DL	U-Net	45	MLS	Acc: 0.94	Deformed right and left hemispheres were automatically segmented. The junction of these two segments was then traced to forms dML.
[43]	DL	U-Net based FCN	38	MLS < 5 mm vs. MLS > 5 mm	Acc: 0.89	MLS estimation at all levels between the lateral ventricles roof and foramen of Monro was performed. The greatest MLS score was considered the final MLS.

**Table 3:** Summary of different AI-based methods for TBI prognostication.

Ref.	ML/DL	Algorithm/Method	Dataset Size	TBI-Related Clinical Assessment	Performance	Main Contribution
[32]	ML	Linear Regression, Binary logistic regression	106	30-day mortality	AUC: Hematoma shape: 0.692 Hematoma size: 0.715–0.786 ICH score: 0.877–0.882 GCS: 0.912–0.922	Hematoma shape and size, ICH score, GCS score, age, IVH, presence of infratentorial location were used to estimate 30-day mortality
[43]	DL	2D U-net based CNN, 3D U-net based CNN	144	Hematoma segmentation, volume estimation, GOSE prediction	Segmentation Dice: 0.697 Volume estimation correlation coefficient: 0.966 6-month GOSE prediction AUC: 0.85	Proposing a novel Multi-view CNN with a mixed loss for hematoma segmentation, quantification, and 6 months mortality prediction
[102]	DL/ML	ANN, LR	785	early mortality	LR Accuracy: 0.87 AUROC: 0.905 ANN ACC: 0.809 AUROC: 0.875	Trauma registry data including Injury, CT findings and demographic characteristics
[103]	DL/ML	LR, 22 ML models *	117	Survival prediction	AUC: LR: 0.83 ML models: 0.30–0.94	Cubic SVM, Quadratic SVM and Linear SVM outperformed LR
[60]	ML	RF classifier	828	6 months mortality prediction	AUC: 0.853 AUPRC: 0.559	Integrating volumetric characteristics and shape features extracted from the proposed model with IMPACT without CT features to predict six-months mortality
[106]	DL/ML	LR, lasso regression, and ridge regression, SVM, RF, GBM, ANN	11022 (IMPACT-II), 1554 (CENTER-TBI)	GOS < 3, GOSE < 5	Mean AUC (external validation) Mortality: 0.82 Unfavorable outcome: 0.77	Motor GCS score, CT class, SDH, EDH, hypoxia, hypotension, demographic, and some laboratory test data were used to compare various model performance in predicting patient outcome.
[104]	ML	XGB, LR	368 TBI patients with GSC<13	Early mortality	XGB: Acc: 0.955 AUROC: 0.955 LR: Acc: 0.70 AUROC: 0.805	Data from electronic medical record system Including laboratory test data, injury, and demographic information, CT finding
[105]	ML	DNN, SNN, LR-net	2164	GOS 1–3 vs. GOS 4–5	AUROC DNN: 0.941 SNN: 0.931 LRnet: 0.919	Using demographic information, GCS, injury mechanism, heart rate, blood pressure and other clinical data
[108]	ML	XGB classifier, SHAP values selection	831 (ProTECT III data set)	GOSE 1–4 vs. GOSE 5–8	AUC: 0.80 Acc: 0.74 F1-score: 0.70	Developing an intelligible prognostic model using 2 rounds of variable selection by SHAP values as well as clinical domain knowledge
[109]	DL	TFNN vs. RF, XGB, SVM	833 (ProTECT III data set)	GOSE 1–4 vs. GOSE 5–8	AUC TFNN: 0.79 RF: 0.80 SVM: 0.79 XGB: 0.74	Developing human interpretable neural network model based on tropical geometry to predict GOSE 6 months after hospitalization

* Quadratic discriminant, Linear discriminant, Fine KNN, Subspace KNN, Coarse KNN, Coarse Gaussian SVM, Medium Gaussian SVM, Fine Gaussian SVM, Cubic KNN, Boosted trees, Subspace discriminant, Simple tree, Medium tree, Complex tree, Cosine KNN, Medium KNN, RUSBoosted trees, Bagged trees, Weighted KNN, Cubic SVM, Quadratic SVM, Linear SVM.

## Data Availability

Not applicable.

## Table’s Abbreviations

DL: Deep Learning, ML: Machine Learning, FCM: Fuzzy c-mean, ICH: Intracerebral Hematoma, EDH: Epidural Hematoma, SDH: Subdural Hematoma, IVH: Intraventricular Hematoma, EAH: Extra-Axial Hematoma, IPH: Intraparenchymal Hemorrhage, Sen: Sensitivity, Spec: Specificity, DSC: DICE, Acc: Accuracy, similarity coefficient, GA: genetic algorithm, FCN: fully convolutional neural network, ICC: Interclass correlation coefficient, NN: Neural Network, CNN: Convolutional Neural Network, RNN: recurrent neural network, RF: Random Forest, GMM: Gaussian mixture model, MDRLSE: Modified Distance regularized level-set evolution, SVM: Support Vector Machine, DRLSE: Distance regularized level-set evolution, AUC: Area Under the Curve, NPL: Natural Language processing, LSTM: Long-Short Term Memory. ICP: Intracranial Pressure, MLS: Midline Shift, GLRLM: Grey Level Run Length Method, DTCWT: Dual Tree Complex Wavelet Transform, DWPT: Discrete Wavelet Packet Transform, iML: ideal Midline, ISS: Injury Severity Score, csfv: Cerebrospinal Fluid Volume, icvv: Intracranial Volume, ABP: Arterial Blood Pressure, FV: Flow Velocity, GA: Genetic Algorithm, SVR: Support Vector Regression, KNN: K-Nearest Neighbors, ONSD: Optic Nerve Sheath Diameter, MAE: Mean Absolute Error, NPL: Natural Language processing, FCN: Fully Convolutional Neural Network, RLDN: Regression-based Line Detection Network, GCS: Glasgow Coma Scale, GOSE: Glasgow Outcome Scale-Extended, LR: logistic Regression, AUPRC: area under the precision-recall curve, XGB: XGboost, RF: Random Forest, GBM: gradient Boosting Machines, ANN: Artificial Neural Network, DNN: Deep Neural Network, SNN: Shallow Neural Network, LR-net: elastic-net Regularized Logistic Regression, SHAP: SHapley Additive exPlanations, TFNN: Tropical geometry-based Fuzzy Neural Network
